# REDCap Delivery of a Web-Based Intervention for Patients With Voice Disorders: Usability Study

**DOI:** 10.2196/26461

**Published:** 2022-03-25

**Authors:** Danielle Mollie Stambler, Erin Feddema, Olivia Riggins, Kari Campeau, Lee-Ann Kastman Breuch, Molly M Kessler, Stephanie Misono

**Affiliations:** 1 Department of Writing Studies University of Minnesota Minneapolis, MN United States; 2 Department of Otolaryngology University of Minnesota Minneapolis, MN United States; 3 Department of English University of Colorado-Denver Denver, CO United States

**Keywords:** web-based intervention, REDCap, voice disorders, usability study, heuristics, eHealth, online, health, web-based participation, patients, web-based platform

## Abstract

**Background:**

Web-based health interventions are increasingly common and are promising for patients with voice disorders because web-based participation does not require voice use. To address needs such as Health Insurance Portability and Accountability Act compliance, unique user access, the ability to send automated reminders, and a limited development budget, we used the Research Electronic Data Capture (REDCap) data management platform to deliver a patient-facing psychological intervention designed for patients with voice disorders. This was a novel use of REDCap.

**Objective:**

We aimed to evaluate the usability of the intervention, with this intervention serving as a use case for REDCap-based patient-facing interventions.

**Methods:**

We used REDCap survey instruments to develop the web-based voice intervention modules, then conducted usability evaluations using (1) heuristic evaluations by 2 evaluators, and (2) formal usability testing with 7 participants, consisting of predetermined tasks, a think-aloud protocol, ease-of-use measurements, a product reaction card, and a debriefing interview.

**Results:**

Heuristic evaluations found strengths in visibility of system status and real-world match, and weaknesses in user control and help documentation. Based on this feedback, changes to the intervention were made before usability testing. Overall, usability testing participants found the intervention useful and easy to use, although testing revealed some concerns with design, content, and terminology. Some concerns were readily addressed, and others required adaptations within REDCap.

**Conclusions:**

The REDCap version of a complex web-based patient-facing intervention performed well in heuristic evaluation and formal usability testing. REDCap can effectively be used for patient-facing intervention delivery, particularly if the limitations of the platform are anticipated and mitigated.

## Introduction

Patients and providers are increasingly turning to digital platforms for medical information and support. The COVID-19 pandemic has further reinforced the need for information and intervention delivery that does not require in-person contact. Web-based interventions are particularly effective for disorders that may impact interpersonal interactions because web-based interventions can reduce barriers to access and communication. One such disorder—vocal dysfunction (or dysphonia)—is common [1] and leads to approximately US $2 billion in annual loss in work productivity [2-4], as well as significant functional and social impairments. Lower quality of life (voice-related) has been reported by patients with lower perceived control [5] (ie, perceived control over events or one’s reactions to events). Perceived control can be increased through targeted web-based intervention [6,7], and greater perceived control is associated with better patient-reported, disease-specific, and overall outcomes such as depression, diabetes, asthma, heart disease, and blood pressure [8-13]. A web-based psychological intervention could thus enhance voice treatment outcomes in a low-cost, accessible way.

Web-based interventions are an especially promising avenue for patients with voice disorders because web-based participation does not require voice use. To be usable by patients, a web-based platform should be Health Insurance Portability and Accountability Act (HIPAA)–compliant, with unique access for users, the ability to send automated reminders, and effective usability. We initially developed a custom website for this purpose, and initial intervention findings were promising [14], but we experienced difficulties related to cost, transparency, and troubleshooting timeliness. Those difficulties were perhaps inevitable, given the limited project resources, and prompted us to explore other potential HIPAA-compliant options for future interventions.

Because our team frequently uses REDCap for data collection, we considered its usefulness for the delivery of a patient-facing web-based intervention. REDCap is an electronic data capture platform widely used in biomedical research because it is secure, HIPAA–compliant, facilitates data exports for analysis easily, and is free or low cost for university researchers under institutional contracts [15]. REDCap’s user-friendly interface reduces the need for programming knowledge and provides additional control through customization options. The platform is well supported, with continuous monitoring, systematic updates, and an increasing list of capabilities that provide a near maintenance-free infrastructure to researchers. Our intervention delivered educational modules over a period of time by sending automated email reminders, for which REDCap’s survey functionality and Automatic Survey Invitation tool seemed well suited. Enabling modules, with respect to timing and sequence, is dependent on multiple inputs, which REDCap is able to capture, calculate, and modify throughout a participant’s use. REDCap functionality also facilitated parallel designs for multiple study arms and simplified study management. We reasoned that the existing functionality might, therefore, be effectively adapted to deliver a patient-facing intervention.

The use of REDCap as a patient-facing intervention is relatively novel. Although REDCap is used by thousands of teams to collect data, it is used far less frequently in a patient-facing manner. The literature does include a few patient-facing studies [16-19], in which REDCap has been used for data collection to assess other (non-REDCap) custom apps or websites. It has also been used for patient interventions as a back-end system paired with custom interfaces such as web pages or interactive forms presented on investigators’ tablet computers [20-23]. However, we found only one study [24] on the use of REDCap for patient-facing intervention delivery and usability.

Given the proliferation of study teams utilizing REDCap and concurrent increasing interest in web-based interventions, we aimed to rigorously evaluate the usability of REDCap for patient-facing intervention delivery in a use case. The objectives of this study were to evaluate the usability of the voice intervention within REDCAP using (1) heuristic evaluation and (2) formal usability testing, which were chosen because they generate complementary forms of usability data. In fact, the combined approach—heuristic evaluation and usability testing—has been described as a “1+2 punch” [25]—providing distinct yet complementary data that can form an excellent baseline for usability. Herein, we also suggest strategies for adapting REDCap to patient-facing health interventions.

## Methods

### REDCap Intervention

**Figure 1:**
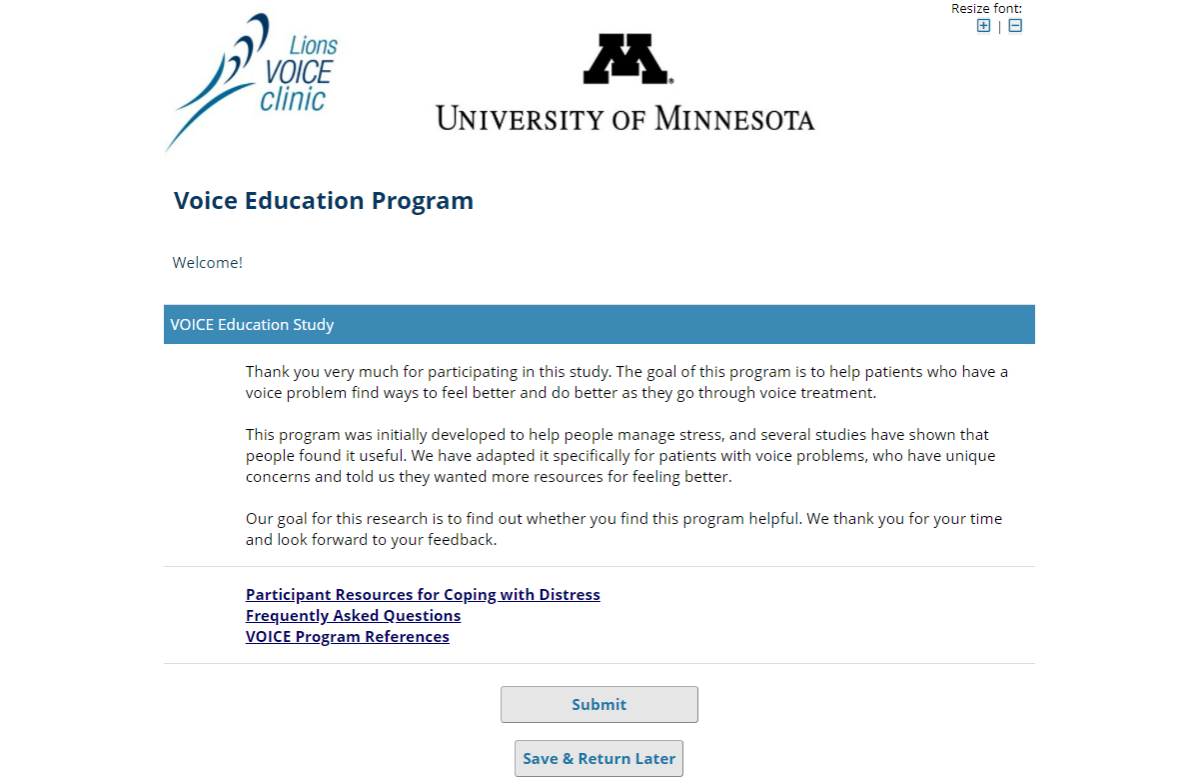
Welcome page for the Voice Education Program intervention.

**Figure 2:**
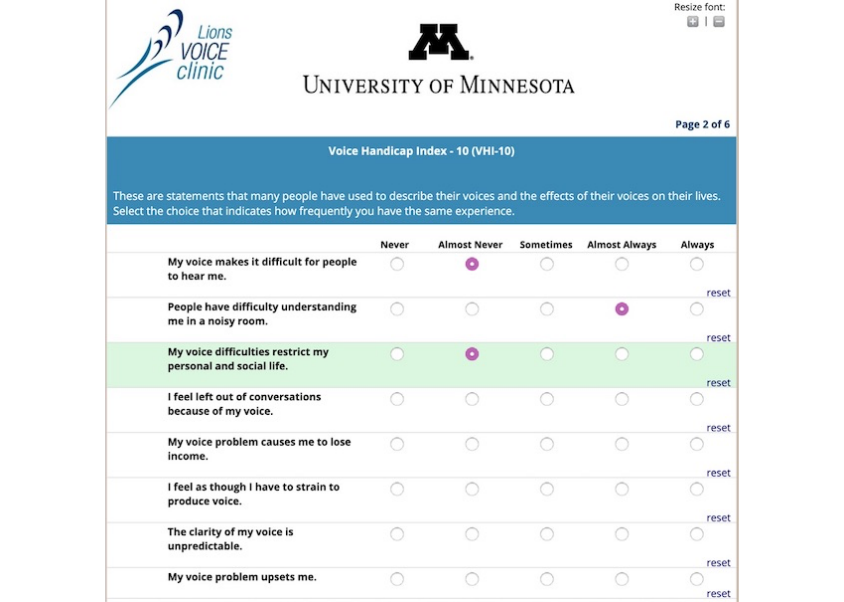
Check-in questionnaire page.

**Figure 3:**
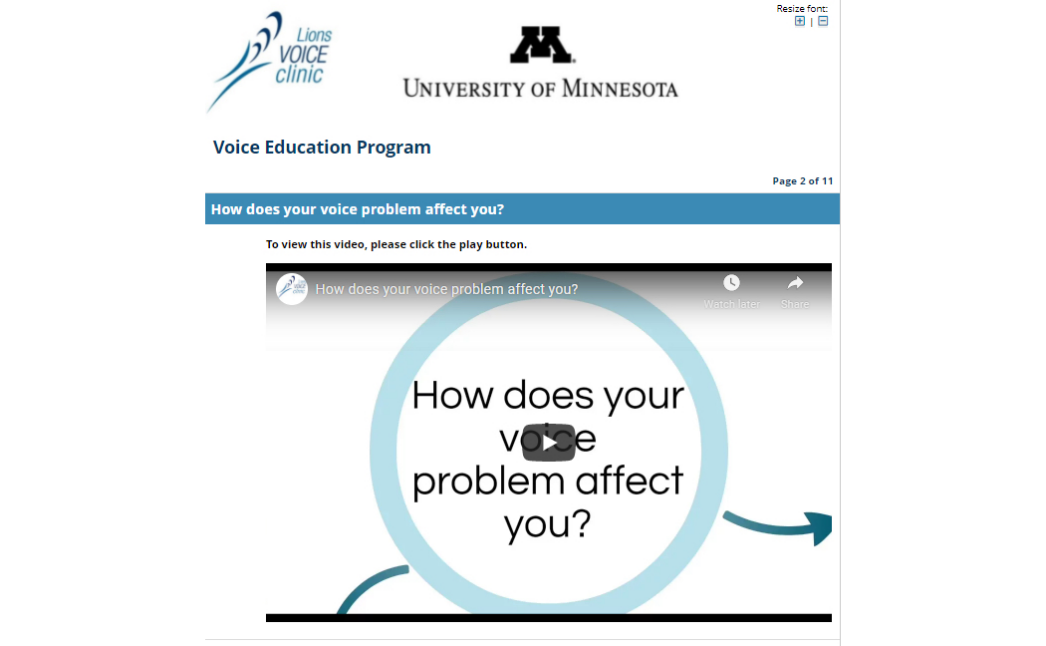
Voice tips page including embedded YouTube video.

The intervention consisted of 3 parts. The first part delivered baseline assessment measures followed by an educational module with instructional videos and self-led exercises. In the second part, participants were invited to complete check-in modules twice a week for up to 3 weeks (Figures 1-3). The third part delivered end-of-study assessment measures (the same as baseline measures) followed by a participation feedback section. The website could adjust total participation time, allowing enough time for the baseline educational module, 2 check-ins, and the final survey module prior to participants starting voice therapy. Individual survey instruments were developed within the REDCap project for each intervention module, and a database instrument was used to set up participant profiles. Conditional logic, using dates and indicator variables manually entered in or captured throughout the intervention, was used to trigger ASIs to alert participants to available modules.

Developing the intervention in REDCap was an iterative process, because REDCap routinely updates functionalities (ie, fixing issues and making desired features possible or easier to implement). The intervention was developed in REDCap (Table 1) with the knowledge that we would later add a comparison arm to be used in a randomized controlled study of the intervention, making use of the randomization tool. The *longitudinal project* setting was tested, but ultimately not used, in this version of the intervention due to limitations of its use with the randomization tool and survey piping features.

**Table 1:** Design parameters used in developing the REDCap intervention.

Category and required design parameter			REDCap features^a^
**Participant access to intervention**			
	Intervention accessible through links sent by email. Reminder emails sent with the deadlines for finishing the module.	REDCap generated unique URLs for each participant when sent by *Automatic Survey Invitation* using *Smart Variables*. Dates were *piped* into the email text.	
	Participants have unique logins to access their information. Participants can save, leave, and return to the website.	Enabled Save & Return in the *Survey Settings*	
	Data must be kept in a secure, HIPAA^b^ compliant database.	REDCap provided a secure web interface and included multiple features to support HIPAA compliance. Both the website and database were housed on secure servers maintained by the researcher’s institution.	
**Intervention design**			
	Educational material able to be delivered by text and videos.	Used *descriptive text fields*, including in-line video	
	Self-led exercises present participants’ prior responses for reflection and goal-setting.	Previous responses were *piped* into descriptive text for participant review.	
	The length of intervention can be shortened if the participant's therapy start date is less than 3 weeks away. Module start and completion dates are otherwise used to enable future modules and trigger emails to send.	Therapy date was entered in the participant set up instrument. Start and completion dates were captured as validated date text variables using the *@HIDDEN-SURVEY Action Tag*. Conditional logic in *Automatic Survey Invitations* using *datediff* calculations and indicator variables sent emails. The *Survey Queue* was used when there was not a lag in time between instruments.	
	Intervention modules are disabled after a period of time to ensure that they are completed in order.	*Time Limit for Survey Completion* option was used in the *Survey Settings*.	
	Hyperlinks to voice and psychological health tips are embedded in the intervention check-ins. Menu and navigation have hyperlinks to resources including the study FAQ, program references, and supplemental mental health resources.	Hardcoded hyperlink embedded in a survey field connected to another REDCap project using the project’s public link. Because a left-hand menu bar was not possible in REDCap and layout was limited to one center panel of text, the menu links were listed in a descriptive text variable at the bottom of each survey page.	
**Study execution**			
	The intervention can adapt to changing participant inputs throughout participant, such as: Updates in contact information. Changing dates of scheduled medical treatment, which affect study participation duration.	A project instrument was used for participant setup to enter dates used for *Automatic Survey Invitations* and manage participant information *piped* into the intervention. An email variable was set as the survey-specific email invitation field in the *Survey Settings* instead of using the *Survey Distribution Tools* to allow for updates over time and ease in participant set up. *Automatic Survey Invitations* were triggered to send the day before the enabling date to allow for date changes during the study, but the researcher could update and retrigger emails if needed using the *Survey Invitation Log*.	
	The intervention moves through modules in sequence.	Default use of the *Survey Queue* allowed linear progression through surveys.	
	Participants cannot go backwards and change answers, which is important for data integrity.	Instrument *Survey Settings* were set to prevent return to and modification of completed responses.	
	Website collects date and time stamps for all responses.	REDCap captured and could report timestamps for survey start, completion, and all responses.	

^a^REDCap-specific terms are in italics.

^b^HIPAA: Health Insurance Portability and Accountability Act.

### Heuristic Evaluation

A heuristic evaluation is a type of inspection method in which web-based interfaces are evaluated based on a list of guidelines for effective interface design. Our team used well-known heuristics [26] that have been used to evaluate both software and web-based interfaces. The goal of heuristic evaluation is to identify areas in which a web-based intervention meets or does not meet widely accepted guidelines for interface design.

Two usability research assistants who were not involved in intervention development conducted the heuristic evaluation. Working independently, the evaluators identified strengths and weaknesses in the intervention’s usability by completing tasks as an end user might, using Nielsen’s usability heuristics for interface design [26].

The evaluators then each generated a report that included specific examples of the intervention’s degree of compliance with each usability heuristic, screenshots of the intervention modules, usability rankings, and rationales for rankings. Strengths and weaknesses identified in both reports were used to update the intervention and guided the development of the usability testing plan and testing scenarios. Each evaluator’s rankings for the intervention’s compliance with the 10 heuristics were standardized on a scale from 1 to 4, with 1 representing ineffective and 4 representing highly effective.

### Usability Testing

In addition to the heuristic evaluation, a usability test was conducted to study the interaction of representative users with the web-based intervention. We designed a usability test around key tasks in the web-based intervention and gathered quantitative and qualitative data to identify successes and problem areas as well as overall participant impressions.

### Setting

Usability testing was conducted on campus. We began usability testing in the Usability Lab on campus, where high-quality data could be collected, including audiovisual recording and screen captures, and where 2 rooms and a one-way mirror provided an optimal research environment for observers. Complications arose in terms of site location—the campus was unfamiliar to most participants and campus sporting events caused disruptions to driving routes and parking availability—therefore, for the last 2 tests, we moved to a research suite closer to the voice clinic familiar to participants, although the suite did not include high-quality data recording or a one-way mirror. We have discussed these challenges in greater detail elsewhere [27].

### Participants and Recruitment

Participants were recruited from MHealth Fairview otolaryngology clinics. Inclusion criteria were diagnosis of a voice disorder; Voice Handicap Index–10 score greater than or equal to 11 [28]; age 18-80 years old; English literacy; and ability to complete informed consent. Because usability testing was completed on campus, potential participants who lived close to the testing site were preferentially invited, although residence location was not used as a strict screening criterion. We recruited 10 participants based on these criteria, and 7 participants completed the usability test.

### Ethics

The study was approved by the University of Minnesota institutional review board (1507S75003).

### Measures, Procedures, and Analysis

Our study’s research questions asked how well patients were able to navigate into, throughout, and exit the intervention; how well patients were able to use multiple choice features to answer questions; and how patients used the *help and documentation* in the FAQ. The usability test consisted of (1) logging into the intervention within REDCap, (2) completing part of a module, (3) reviewing supplementary material and navigating back to a module, (4) exiting REDCap and logging back in, and (5) identifying the help page within the intervention (Multimedia Appendix 1). A moderator facilitated each usability test and asked participants to complete each task using a think-aloud protocol (in which participants share their thoughts as they work through the task [25]). An additional research question, which focused on understanding patient experiences with the intervention, was added during usability testing [27].

Usability measures were time-on-task, task completion rates, issue rates and severities, and subjective user satisfaction. Time-on-task reflected how long it took a participant to complete a task from the time it was given until the time the participant indicated completion. Our goal with time-on-task measures was to establish a realistic baseline time, thus we did not set specific target times. Task completion rates were measured as the percentage of test participants who were able to successfully complete the task without requiring assistance or encountering high-severity issues. Our goal for task completion was 100%. After each usability test, we counted issues and rated each for severity; a high-severity issue (a severity rating of 1) prevented a participant from correctly completing a task, while a low-severity issue (a severity rating of 5) did not change the outcome of the task but resulted in the task being completed less efficiently. Our goals for issues per participant were less than 1 high-severity issue, less than 5 moderate-severity issues, and less than 5 low-severity issues. Additionally, we ranked issues based on their frequency across participants, with low-impact but high-frequency issues overall being rated at a higher severity level.

User satisfaction was measured by asking participants to rate ease of use for each task on a scale of 1 to 5, with 1 representing very easy and 5 representing very difficult. Our goals were to have no posttask user satisfaction rating higher than 3 for any individual participant, and an overall average rating of 2 (easy) for each task across all participants. After each task, the moderator invited participants to offer any comments about the rating they chose.

After each participant completed all tasks, we asked them to complete a product reaction card and debriefing interview describing their experience. The product reaction card was a sheet with a set of 63 positive and negative words from which participants were asked to choose 5 that best described their experience [25]. This set was derived from a desirability matrix [29] of 118 words, with a ratio of 60% positive to 40% negative or neutral words [25]. The matrix can be used in full or abbreviated to gather quick descriptive feedback about participant impressions [30]. Our goal was to have at least 60% of all reaction words be positive. Debriefing interviews included 5 open-ended questions asking participants to describe their initial impressions, how those impressions changed as they used the intervention, what they liked least and best, and what they would change if they could (Multimedia Appendix 2). In combination with the think-aloud protocol, the debriefing interview allowed for insights into participants’ health care-related contexts of use and engagement with intervention content [27].

## Results

### Heuristic Evaluation

The heuristic evaluation (Table 2) indicated that 3 categories could be improved: user control and freedom, consistency and standards, and help and documentation. These 3 areas of improvement were used to make initial revisions to the intervention and also informed the creation of usability testing tasks and questions.

**Table 2:** Heuristic evaluation results.

Heuristic	Rating	Strengths	Areas for improvement
1. Visibility of system status	3	System showed current status effectively in main survey sections via page counts, color confirmation, and written confirmation	System status and future options were not as apparent in additional help sections
2. Real-world match	2	Survey section numbering, sequence, and naming were logical and consistent with real world conventions Survey questions follow conventions for type and format Embedded YouTube videos take advantage of familiar features, platform	Procedure for leaving and returning was not conventional or natural “Survey” terms in standardized research questionnaires did not match real-world conventions Additional section links are hard to find, and function in unconventional and nonnatural ways
3. User control	2	Reset function was an effective undo feature	“Emergency exits” were unclear in additional help sections
4. Consistency	2	Main survey sections used consistent layout, functioning, color, and terms	Pop-up boxes, formatting, headers, and tone were inconsistent within and across pages
5. Error prevention	3	Several effective error prevention features (eg, prohibits leaving questions unanswered)	No prevention against accidentally closing whole survey window without saving
6. Recognition	3	Instructions for system use are readily available throughout Questionnaires and check-ins provide built-in references to past information Educational videos, FAQ^a^, and additional resources are available at the bottom of each page	Contents and options are not centrally listed in the additional vocal health tips sections
7. Flexibility	3	Font resize option is available Survey queue automatically accordions up as surveys are completed, but still provides an option to view all	Menus with links to additional resources and vocal health tips sections cannot be hidden
8. Aesthetic	3	Aesthetic is simple, neutral, and uncluttered	Some images in the additional vocal health tips sections are less relevant and therefore less impactful than they could be
9. Error messaging	3	Error messaging is clear and provides both an explanation and a solution	None identified
10. Help and documentation	2	FAQ and help email are readily available on survey queue/home page FAQ is available on all main survey pages Instructions for use are available throughout the module	Help is not searchable No documentation for technical issues No centralized overview of instructions, features, problems, and complex tasks

^a^FAQ: frequently asked question.

Heuristic evaluation indicated that visibility of system status, real-world match, and recognition were all intervention strengths, aligning with REDCap’s ability to provide a stable platform that matches users’ expectations for websites, without requiring any specialized development knowledge to build and maintain.

The heuristic evaluation indicated user control and freedom, consistency and standards, and help and documentation as 3 major heuristic categories in need of improvement. Of these weaknesses, the category user control and freedom was most pertinent to REDCap’s functionality as a platform. Consistency and standards, as well as content in help and documentation, were readily addressable once identified. This included following recommendations for plain language [31] and ensuring parallel structure. REDCap allowed for immediate updating of all edited content without the need to rely on a third party for content editing.

### Usability Testing

A total of 10 participants were recruited; 7 participants completed the usability test. All participants were patients at MHealth/Fairview with voice problems (Table 3).

**Table 3:** Demographic characteristics of study participants.

Characteristic			Value
Age (years), mean (range)			51 (30-71)
**Gender (n=7), n (%)**			
	Male	1 (14)	
	Female	5 (71)	
	Gender nonconforming	1 (14)	
**Race/ethnicity (n=7), n (%)**			
	White	5 (71)	
	African American	1 (14)	
	Asian American	1 (14)	
**Education (n=7), n (%)**			
	Some college credits, no degree	1 (14)	
	Bachelor’s degree	1 (14)	
	Graduate degree	5 (71)	
**Used web-based health resources before (n=7), n (%)**			
	At least once a week	2 (28)	
	At least once a month	1 (14)	
	Less than once a month	3 (43)	
	Decline to answer	1 (14)	

**Figure 4:**
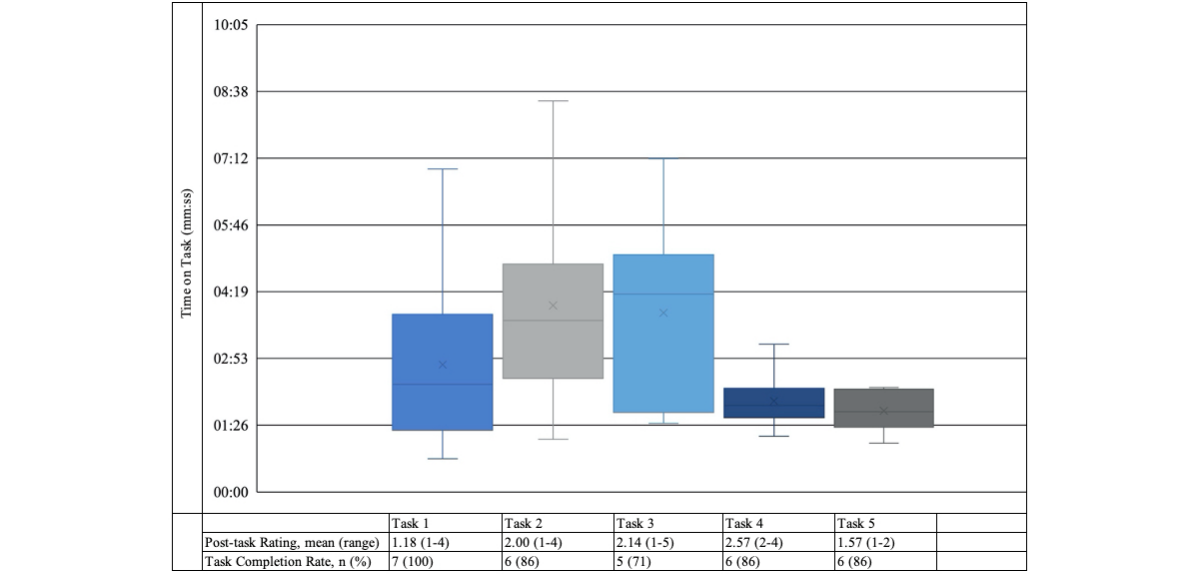
Time-on-task, mean post-task ratings, and task completion rates.

Results from the usability tests include time-on-task, task completion rates, issue rates and severity, product reaction card selection, and qualitative data from debriefing interviews. Task completion rates ranged from 71% (5/7) to 100% (7/7). Overall, mean posttask ratings were close to our goals, and the individual highest posttask ratings exceeded goals on 4 of 5 tasks (Figure 4).

Issue rates met specified goals for all but 1 participant, who experienced several critical issues; only 1 issue—the use of the *Submit* button—reflected an issue both high in frequency and severity. Four other issues were noted as high impact but with low severity (Table 4).

**Table 4:** Summary of usability issues grouped by topic.

Category and issue			Participants encountering issue, n (%)		Overall frequency		Individual severity ratings		Overall assessment of severity
**Navigation**									
	Did not realize that the *Submit* button on the landing page was for moving forward in the survey	4 (57.1)		High		1, 3, 3, 4		Critical, high impact	
	Unclear how to enter first survey	1 (14.3)		Low		1		Critical, high impact	
	Clicking the back button resulted in an error message	2 (28.6)		Moderate		1, 2		Critical, high impact	
	Unclear how to return to the intervention after logging out	1 (14.3)		Low		2		Noncritical, moderate impact	
**Intervention features**									
	Small browser window caused text to wrap, which was difficult to read	1 (14.3)		Low		3		Noncritical, low impact	
	Unable to locate health tips	1 (14.3)		Low		1		Critical, high impact	
**Help and documentation**									
	Unsure if FAQ^a^ was the right place to look for help	1 (14.3)		Low		4		Inconvenient, lowest impact	
	Identified a different page as the FAQ	2 (28.6)		Moderate		3, 4		Noncritical, low impact	
	Would try to contact MyChart (a clinical system) for help	1 (14.3)		Low		1		Critical, high impact	
**Content**									
	Text-heavy or wordy pages	4 (57.1)		High		4, 4, 4, 4		Inconvenient, lowest impact	
	Discomfort with psychological questions	5 (71.4)		High		3, 3, 4, 4, 4		Noncritical, low impact	

^a^FAQ: frequently asked question.

Debriefing interviews revealed generally positive first impressions of the interface, and these positive impressions persisted throughout the test. Participants’ dislikes and recommended changes were aligned with the usability issues documented during testing. When asked to comment on their first impressions of the intervention, 5 participants reported positive first impressions, of whom, 3 participants focused specifically on the visual impression of the interface and noted that it “look[ed] clean,” appeared “pretty straightforward,” and was “nice, clear, easy to read.” Two participants reported negative first impressions and focused on the text-heavy nature of the homepage. Three participants noted that the *Submit* button made entry to the intervention somewhat confusing. Participants all reported that their first impressions did not substantially change as they navigated the intervention.

When asked what they liked best about the intervention, 5 participants reported that the resources and content were “informative,” “useful,” and “helpful,” and 4 participants spoke to the design of the intervention and said that they appreciated how the content was “succinct,” “clearly written,” “not overwhelming,” and that the fonts and color schemes were “pleasant.” When asked what they liked least about the intervention, 3 participants reported that there was nothing they did not like, 3 participants did not like the wordiness, and 1 participant described it as a “general sense of clutter.” One participant would have liked “prettier colors,” and 2 participants did not like the questions that the interface posed about their voices: one participant wanted to know who would read and respond to her responses to these standard questions, and the other participant did not look forward to having to “write a lot of stuff out about my voice...that's not enticing.”

When asked what they would change about the intervention, 2 participants said that there was nothing they would change. The other 5 participants recommended changes such as adding video content; providing short summaries of the content on each page; less text, especially on the front page; changing the *Submit* button on the landing page; and incorporating more consistent branding, such that the intervention would be more clearly connected to the clinic.

In participants’ responses to the product review card, 89% of reaction words were positive. Only 1 participant chose negative words. Despite posttask ratings and observed issues that reflected more difficulty than we expected (Figure 4 and Table 4), participants’ word choices on the product reaction card indicated positive feelings about the intervention. Out of 7 participants, 5 participants chose the word “organized,” and 4 participants also chose “convenient” and “easy to use.” Other common choices were “helpful,” “relevant,” and “useful.” Although, it may seem that product reaction card results conflict with posttask ratings and observed issues, results from the debriefing interviews supported an overall positive participant reaction to the intervention. Participants were highly engaged with the content itself and liked that it was web-based, although that was not the target of usability evaluations.

Overall, although participants experienced some challenges when completing tasks within the intervention, all usability metrics met or exceeded our predetermined goals regarding task completion, posttask ratings, and positivity of intervention descriptive words.

## Discussion

### Principal Findings

Our heuristic evaluation and usability test results provide evidence that REDCap is a useful platform for patient-facing web-based information and intervention delivery. Our findings demonstrate that the REDCap intervention was functional and usable for participants. Participants’ comments demonstrated that they found the intervention and, by extension, its REDCap interface, to be one that they could imagine layering into their existing medical routines. Because the use of the REDCap platform for direct interaction in a patient-facing health intervention is relatively novel, and to the best of our knowledge, this is the first formal usability assessment of REDCap used in this manner, below we delineate specific recommendations for researchers wishing to develop REDCap for delivery of patient-facing interventions.

### REDCap Development Recommendations

We found that the successful conversion of our initial custom website to a patient-facing web-based REDCap intervention aligned with and supported the overall user-friendliness of REDCap for investigator use. Our novel use of REDCap was further supported by the overall high acceptability and usability observed in our formal testing. We did encounter specific challenges, and additional information on strategies that proved useful for our team is offered below for others considering similar approaches to patient-facing information or intervention delivery. Some of these challenges and mitigating strategies may become obsolete as REDCap functionality is continually updated. For example, in a recent update, a data dictionary was created for ASIs, which greatly improved the ease of intervention development and troubleshooting. In addition, REDCap is developing options such as Mobile App and MyCap for use on mobile devices [32].

Our study also highlighted specific and persistent features of the REDCap intervention that detracted from user experience. Navigation challenges identified in heuristic evaluations—moving between help and survey queue pages, and finding “emergency exits” from the modules—suggest that REDCap is currently best suited to interactions that do not require extensive navigation between different modules. Additionally, while REDCap’s error messaging was clear and timely, there were fewer options for making help documentation readily available throughout the intervention without a workaround such as a link menu at the bottom of the page. When we evaluated the issues that participants experienced when completing the usability test tasks, we found that some issues were related to the structure and limitations of REDCap as an interface. Thus, our usability findings also underscore the value of insights gained from research with end users and could help other researchers deploy REDCap as a patient-facing intervention delivery method.

We encountered a few issues in converting a custom website to a REDCap format; some issues remained unresolved and were tested in the usability evaluations. Issues highlighted by usability testing fell into 3 categories: conceptual expectations of website, nonintuitive navigation, and confusing site architecture (Table 5).

**Table 5:** Adaptations in REDCap to address heuristic and usability findings.

Challenges with REDCap^a^ intervention delivery			Sample adaptation to enhance usability
**Mismatch between format and the conceptual expectations of a website**			
	Heuristic analysis recommended better distribution of white space by moving information to the footer, header, or side menus where possible.	Participant resources links and survey page instructions were moved to the Survey Footer to separate them from module related text.	
	Participants recommended more branding visibility, as they appreciated the affiliation of the project with their clinic.	Aesthetics were constrained by limited options where logos could be added; combined logos were created to allow multiple entities to be represented.	
	Participants found page titles confusing and recommended clearer instructions and wording about the intervention and module titles.	Headers and text were revised and simplified to clarify instructions about the study and intervention and make the intervention consistently identifiable on each page.	
**Nonintuitive navigation through the program**			
	No independent home page functionality besides using the *Survey Queue* as a starting point, which participants found unfamiliar and confusing.	A site map was not possible within REDCap. Therefore, study status graphics were added to the first and last page of each module to show the participant’s progression through the intervention.	
	Participants struggled to tell how far along they were in the program, as the survey queue did not show what was forthcoming when using *Automatic Survey Invitations*.	Page numbers were added to show progression through each module.	
	Participants found that saving and returning using the randomly generated code for re-entry was nonintuitive and easy to miss when leaving a survey, making returning to the intervention difficult.	The *Survey Login* was enabled to use participant email to log into REDCap instead of a random generated code. Instructions for navigation in the FAQ^b^ were added and linked to the FAQ in the *Automatic Survey Invitation* email(s).	
	Participants experienced difficulty returning to REDCap intervention pages after clicking on a hyperlink due lack of ability to link back to other instruments within a survey.	The number of embedded hyperlinks was minimized. Where hyperlinks were unavoidable, instructions were added, eg, how to navigate back to the next part of the intervention from the patient resources webpage.	
**Confusing site architecture**			
	REDCap’s participant-facing interface was the survey format, and participants struggled with hardcoded survey labels and buttons such as *Survey Login* or *Close Survey*.	Instructions were revised to say “survey” instead of “assessment” or “questionnaire.” When removing survey labels was not possible, such as for instructions in the linked tips and help documentation, descriptive text with instructions was added, eg, “Click ‘Close Survey’ to close this window. Then go back to the program page.”	
	Using the *Survey Queue* as the home page for the intervention confused some participants because the program was not a survey in the typical sense.	Visible use of *Survey Queue* was replaced with study status graphics at the beginning and end of each module to limit the amount of “survey” titles and buttons. The *Stealth Queue* external plug-in was used to prevent the survey queue from automatically displaying at the end of a survey.	
	To advance, participants needed to click the *Submit* button, even if nothing was being submitted, such as after viewing educational material.	The number of instruments per module was reduced to limit the number of *Submit* buttons.	

^a^REDCap-specific terms are in italics.

^b^FAQ: frequently asked question.

### Strengths and Limitations

Key strengths of this study were the multidimensional nature of our assessment, with both heuristic evaluation and formal usability testing. For the latter, we incorporated objective and subjective task-based data, such as timing and scoring, as well as open-ended data formats, such as think-aloud responses and a debriefing interview. This layered structure allowed for a rich examination of multiple types of usability data from the patient-facing REDCap intervention. Another key strength of the study is that the team was multidisciplinary and included expertise in usability, writing studies, engineering, psychology, voice, and medicine, which allowed the incorporation of perspectives from multiple areas, which in turn, strengthened the potential generalizability of findings. In addition, the study was completed with patients from the target population for the intervention, which increased the face and content validity of our findings [33]. Perhaps the greatest strength of this study is that it offers a practical approach to a challenging problem: how to translate helpful content into a format that is usable for patient participants in an affordable, transparent manner. Our findings allow for ready expansion to create comparison arms for our existing studies and would be useful to other research teams pursuing similar avenues of investigation and to clinicians or others who may wish to deliver information to patients and clients in an interactive secure manner. The limitations of the study include its small sample size and the limited diversity therein, which both impact generalizability. However, the strengths of the study outweigh its limitations, and we hope to address these limitations in future studies.

### Considerations for Researchers

REDCap is an appealing platform for a web-based intervention because of its ease of use for researchers and participants, favorable cost and accessibility, and overall effective usability. Furthermore, REDCap is an evolving resource, with additional functionalities frequently being added. In some cases, new functionalities alter the behavior of current active projects, and in other cases, new functionalities offer helpful solutions to important challenges. We recommend that researchers developing an intervention with REDCap’s current capabilities consider customizing REDCap delivery based on intervention needs using tools such as field variables, structured module timing, and piping options; minimizing the use of tools that display the hardcoded term “survey” in the text, such as survey queue, submit survey buttons (unless the study is purely a survey); enabling survey log-in and provide clear information on how to navigate in and out of the intervention; making stage of progression through the intervention clear (eg, page numbers, study status graphic); and paying close attention to REDCap updates that may change functionality.

REDCap may be particularly helpful for developing functional intervention prototypes, because it allows researchers to efficiently incorporate changes based on participant feedback for rapid testing of iterations of the content and format. It also allows for the efficient creation of comparison study arms. Interventions developed in this manner could be optimized and permanently used in REDCap, or used as a functional prototype or model for a custom website. Overall, our findings suggest that REDCap can effectively be used for patient-facing intervention delivery, particularly with adaptations such as those suggested above to optimize its usability. We anticipate that, as REDCap evolves and continues to partner with clinicians and researchers, its applicability will expand even further, reducing barriers for teams offering patient-facing interventions.

## Appendix

Multimedia Appendix 1 Usability test tasks.

Multimedia Appendix 2 Debriefing interview questions.

## Abbreviations

ASI: Automatic Survey Invitation
FAQ: frequently asked question
HIPAA: Health Insurance Portability and Accountability Act
REDCap: Research Electronic Data Capture
