# Supplementary material for: REDCap Delivery of a Web-Based Intervention for Patients With Voice Disorders: Usability Study
Source: JMIR Hum Factors. 2022 Mar 25;9(1):e26461. doi: 10.2196/26461 (PMC8994149; doi:10.2196/26461)
Supplement: Multimedia Appendix 1 [file humanfactors_v9i1e26461_app1.docx]

**Usability Test Tasks**

We created tasks related to the research questions. The following tasks were used in the usability test:

- Task 1 : Login, stop. “You have opened an email to start the Voice Education Program. You want to complete Voice Check-in 1. From your email, go to the VOICE site and log in. Say ‘done’ when you are finished.”
- Task 2: Open questionnaire, stop at page four. “You need to describe your voice and the effects your voice has on your life. Use Voice Check-In One interface to do so. Stop when you get to the beginning of page 4. Say ‘done’ when you are finished.”
- Task 3: Review vocal health tips, navigate back to module. “You want to review some vocal health tips, then continue finishing the module’s questionnaire. Explore a voice-related tip category in the links. When you have explored a tip, then navigate back to page four of the check-in questionnaire. Say ‘done’ when you are finished.”
- Task 4: Exit the system and log back in. “You would like to take a break and come back to finish later. Exit the system completely, close your browser, and then log back in. Say ‘done’ when you are finished.”
- Task 5: Getting help. “You are having issues with the website. You decide to look for help options. Say ‘done’ when you are finished.”
