# Supplementary material for: REDCap Delivery of a Web-Based Intervention for Patients With Voice Disorders: Usability Study
Source: JMIR Hum Factors. 2022 Mar 25;9(1):e26461. doi: 10.2196/26461 (PMC8994149; doi:10.2196/26461)
Supplement: Multimedia Appendix 2 [file humanfactors_v9i1e26461_app2.docx]

**Debriefing Interview Questions**

After all tasks in the test, the moderator conducted a debriefing interview. The following questions were included in the debriefing interview:

1. What was your first impression of the module?
2. Did that impression change as you used it, and if so, why?
3. What did you like best about the pages where you did the test tasks, and what did you like least?
4. If anything, what would you change about the pages where you did the test tasks?
5. Do you have any additional comments about your experience?
